# On the Efficacy of Indigenous Knowledge Systems in Responding to the COVID-19 Pandemic: Unsettling Coloniality

**DOI:** 10.3390/ijerph21060731

**Published:** 2024-06-05

**Authors:** Jabulile H. Mzimela, Inocent Moyo

**Affiliations:** Department of Geography and Environmental Studies, University of Zululand, KwaDlangezwa 3886, South Africa; moyoi@unizulu.ac.za

**Keywords:** indigenous knowledge systems, COVID-19, decoloniality, Africa, uMkhanyakude district municipality

## Abstract

Indigenous groups across Africa mobilized Indigenous Knowledge (IK) practices, albeit not without challenges, to respond to the COVID-19 pandemic. Yet Indigenous Knowledge Systems (IKS) continue to be sidelined in formal healthcare policies and programmes. This underscores the urgency to liberate Africa’s epistemologies. Employing the decoloniality lens, this paper examined the colonial influences inherent in African responses to COVID-19 while also exploring the role of IKS in the uMkhanyakude District Municipality (UKDM). The argument is made that, in the case of the UKDM, the efficacy of IKS was demonstrated in the response to and fight against the COVID-19 pandemic. This is the basis for the call to embrace and recognize that IKS is a legitimate body of knowledge comparable to Western science. Such recognition paves the way for more equitable, contextually relevant, and sustainable health strategies that can better address the complexities of current and future pandemics.

## 1. Introduction

The emergence of the novel Coronavirus disease in 2019 (COVID-19) had a profound and unparalleled impact on the global community, leading to its classification as a global pandemic by the World Health Organization (WHO) on 11 March 2020 [1]. This health crisis presented multifaceted challenges across local, national, regional, and international levels, testing the readiness and capability of various entities to effectively prepare for and counter its effects [2]. Tragically, the pandemic resulted in the loss of lives while straining public health systems and triggering extensive socio-economic disruptions, disproportionately affecting the most vulnerable populations [3]. Despite the plurality of knowledge on healthcare, the predominant influence on policy and programmes in response to the COVID-19 pandemic worldwide remained Eurocentric. This stemmed from the dominant Eurocentric scientific paradigm legitimized by governance structures, the market, and professional experts [4]. The effect of this has been the marginalization and/or peripheralization of diverse Indigenous Knowledge Systems (IKS). This was the case even in Africa due to the enduring coloniality [5] and the lasting repercussions of apartheid in South Africa [6]. These historical events have engendered a legacy of exploitation, uneven power dynamics, and cultural disruption, persistently shaping contemporary realities.

The COVID-19 pandemic has underscored the persistent structural violence directed towards IKS and other non-dominant forms of knowledge. IKS, as a concept, remains a subject of scholarly discourse; however, it is broadly recognized as a “body of knowledge built through generations living in close contact with nature, and it includes a system of classification, a set of empirical observations about the local environment, and a system of self-management that governs resource use” [7] (p. 2). The hegemony of Eurocentric philosophical conceptions of knowledge reinforces the notion that Africans are inferior to former colonizers and undermines and marginalizes African values and IKS [8] because non-Western knowledge systems are appropriated or devalued [9]. IKS have been sidelined in the official framework of COVID-19 responses, particularly by global and national governance structures. However, the pandemic prompted communities to look beyond government-driven Western approaches, which proved inadequate in responding to the crisis. In other words, communities were forced to draw on expertise from IKS to treat or prevent infection. This was particularly pertinent considering the challenges plaguing the global distribution of vaccines, including issues of equity, logistical complications, and the emergence of new outbreaks and variants [3]. 

IKS have been instrumental in bolstering the resilience of African communities against health crises, serving as a foundational resource throughout various historical periods, including during and after the dissolution of colonial empires. This historical context highlights how African societies have adeptly navigated a range of epidemics and pandemics, employing IKS as a crucial tool for community health and survival. For example, Africa suffered from the influenza pandemic in 1968, the avian influenza A subtype H5N1 outbreak in 2003, and the spread of influenza A subtype H1N1 in 2009 [10]. Insights gleaned from these influenza pandemics, along with experiences from tackling other epidemics like HIV/AIDS (in the early 1990s) and the Ebola virus (in the mid-2010s), were swiftly adapted to enhance Africa’s resilience in the face of the COVID-19 [2]. That is, Africa’s enduring history of countering epidemics through the utilization of “condemned” IKS has proven to be effective in confronting the challenges posed by COVID-19 across the continent [5,11]. African communities have developed unique approaches to symptom identification, disease prevention, and treatment, which are deeply rooted in generations of empirical knowledge and practice [12]. The strength and significance of African epistemologies lie in their inherently experiential and experimental nature [5].

Africans were not formally afforded the opportunity to tailor their response to COVID-19, as the approach to the COVID-19 response was imposed upon them. The imposition of a one-size-fits-all strategy, driven mainly by external entities, overlooked the intricate fabric of African societal norms, including cultural traditions, social structures, livelihood strategies, communal practices, and gender dynamics. This oversight effectively marginalized the interests and welfare of Africans. That is, the interests of Africans were ignored. While there is emerging literature in South Africa regarding the role of IKS in responding to COVID-19, it remains in its early stages of development. As a result, a significant gap persists, prompting this paper to adopt a decoloniality lens to examine the colonial influences inherent in African responses to COVID-19 while also delving into the role of IKS in shaping these responses. The goal is to illustrate the efficacy of IKS in healthcare policy within the context of the UKDM despite the fact that it is being silenced and depoliticized. Such an analytical position uncovers the colonial underpinnings of African responses to COVID-19 but, at the same time, illuminates the role and relevance of IKS in an African context, and this should be the basis for more just and contextually attuned strategies for preparing for and addressing pandemics. To pursue this argument, the next section discusses decoloniality, followed by a brief analysis of the politics of knowledge production. Following this, attention shifts to the selected study area and an outline of the methodological approach undertaken for data collection and analysis. This is followed by a section that analyses the data. In the concluding section, the point is made that IKS are legitimate, with the same standing as Western science, and this should be the basis for transcending the latter as the only science that can be used to respond to pandemics like COVID-19. This paper contributes to the discourse on decolonizing health responses, advocating for a future where health interventions are informed by a broader spectrum of human knowledge and experience.

## 2. On Decoloniality

Decoloniality is among “many others against the ongoing violence, dispossession, and war altogether waged against specific bodies, against people’s cultures, knowledge, spiritualities, and against nature, and for the insurgent and resurgent creation, construction, and possibility of other modes of that knowledge being existence in life” [13] (p. 1). In this vein, decoloniality “resists or is against colonial domination. It is not just resistance or a posture of resistance, but it’s for the ongoing creation of ways of thinking, of ways of knowing, of ways of sensing, being, and living outside coloniality. Outside or despite coloniality, and in its borders, its fissures, and its cracks. So, in that sense decoloniality necessarily brings forth or points to the issue of coloniality” [13] (p. 1). At the “centre of decoloniality is the idea of remaking the world such that the enslaved, colonised, and exploited peoples can regain their ontological density, voice, land, history, knowledge and power” [14] (p. 23). Taken further, decoloniality “can be best understood as a pluriversal epistemology of the future—a redemptive and liberatory epistemology that seeks to de-link from the tyranny of abstract universals” [15] (p. 5). This is all important because “decoloniality is distinguished from an imperial version of history through its push for shifting of geography of reason from the West as the epistemic locale from which the ‘world is described, conceptualised and ranked’ to the ex-colonised epistemic sites as legitimate points of departure in describing the construction of the modern world order” [15] (p. 13). In all this, it is important to emphasize that decoloniality “is not a singular theoretical school of thought but a family of diverse positions that share a view of coloniality as the fundamental problem in the modern age” [15] (p. 13). The relevance of decoloniality as a theoretical framework in this paper is that it assists to question responses to the COVID-19 pandemic. This, by highlighting the coloniality of the said responses, then provides the basis of how and why this must be corrected to put at the centre stage IKS as a legitimate body of knowledge to tackle pandemics like the COVID-19. Decoloniality views the distribution of material and natural resources, risks, impacts, and access to environmental decision-making as reflecting coloniality and socio-political inequality. Coloniality determines access to specific forms of knowledge, space, resources, and socio-political processes [5]. This calls for politicized investigations of the response to pandemics like COVID-19, which cannot be understood through scientific research alone. Such pandemics result from interactions between socio-technical politics and institutional structures, including dynamics related to social identities (e.g., gender, class, and religion, among others). For example, exposure to COVID-19 was relatively uniform in the UKDM. However, colonialism-related power disparities and resource access and control determine vulnerability and response, reinforcing social marginalization and inequality.

In this sense, decoloniality assists in examining and laying bare issues of inequality and power in the COVID-19 response. The intention is to expose tenacious power structures to locate and explain the origins and causes of marginalization, injustices, and vulnerabilities in response to COVID-19, thereby avoiding a romanticized view of the global response in the face of socio-cultural variety. This is because the South African government’s policies were based on Western epistemologies. This implies that power and politics are at play, which impedes the mobilization of IKS. Bridging this research gap yields novel insights for addressing the COVID-19 response at the intersection of institutionalized power inequality and politics. In turn, this will benefit policy and programme development decisions.

## 3. Politics of Knowledge Production and Medical Colonialism in Africa

In this section, we discuss the nature of epistemic injustice facing IKS. We delve into the perplexing question of why we persist in retrofitting Western solutions onto African contexts. We examine the critical questions of “Whose knowledge carries weight?” and “Whose knowledge confers expertise?” and “Whose knowledge is recognized at all?” in response to the COVID-19 pandemic. The global community was ill-equipped to confront the pandemic. Hence, multiple epistemologies should have been considered to strengthen resilience and not just Western conceptions, while IKS faced epistemic injustice. IKS receive different patronage from governance structures, the market, and health professionals, hence the hierarchization of knowledge in global healthcare. This hierarchy dictates the selection of knowledge for translation regarding policy and programmes [4]. Over generations, Eurocentric modernity has systematically excluded IKS from development agendas [16], making Europe and North America the primary repositories of knowledge. This exclusion has denied the validity of non-dominant knowledge, such as IKS based on the untested view that the safety, efficacy, and quality of IKS cannot be validated like scientific knowledge using theoretical and methodological approaches. As a result, the vaccine was presented as a panacea [4], and IKS were disregarded. This does nothing for the decolonization of knowledge. Moreover, the question arises as to who holds the authority to decide on the assessment measures for the validity of knowledge [4]. 

Several reasons have been proposed for the neglect of IKS, including the perception of IKS as being of lesser value and legitimacy than Western scientific knowledge; lack of/poor accessibility of IKS to policy-makers due to lack of documentation; and the static nature of IKS, which lack a feedback mechanism to adapt to external influences [17]. Moreover, some underlying IKS belief systems are incompatible with capitalist economic systems [18]. WHO observed that about 80% of people in developing countries use traditional medicine [6]. However, the WHO protocols and the regulations stipulated by the Coronavirus National Command Council of the South African government predominately translated knowledge based on Western conceptions without due care for contextual relevance and sidelined indigenous healers [6]. An appeal arose for the acknowledgement and integration of traditional healers within the South African healthcare system [6]. Despite this, IKS was castigated and perceived as primitive, guilty, and inadequate to contribute to the fight against the pandemic [6,12]. These sentiments align with the assertion made by Ndlovu-Gatsheni [19] (p. 373) that “geopolitics of power and knowledge are still tilted toward the Global North as the only site of credible science.” This necessarily speaks to the issue of the coloniality of knowledge, which concerns “the politics of knowledge generation as well as questions of who generates which knowledge, and for what purpose” [15] (p. 11). Decoloniality of knowledge allows one to question why Europe and North America are the centres of knowledge leading to the rest of the world including Africa and its people being regarded as and reduced to subhuman people without knowledge [19,20]. This is equivalent to wiping away IKS and replacing them with Eurocentric knowledge. This invasion of the mental universe has been characterized as the “removal of the hard disk of previous endogenous knowledge and downloading into the African minds of the software of European knowledge and language” [19] (p. 8). This amounts to the Europeanization of the world and subalternation of the rest of the world including Africa [20]. 

This effectively means that the process of translating knowledge is inherently entangled with political dynamics. The emergence of the COVID-19 pandemic has intensified the existing power struggles concerning knowledge between the Global North and the Global South [19]. However, a fundamental question arises: Why should the act of translating knowledge result in the systematic devaluation of particular knowledge forms in favour of others [4]? Despite having previously grappled with epidemics and pandemics, Africa has witnessed a reluctance on the part of governance structures to tap into this knowledge repository for crafting responses to the COVID-19 crisis [5]. African therapies are widely used across the continent for healthcare due to their accessibility, cost-effectiveness, and cultural acceptance [21]. Incorporating different knowledge systems in the COVID-19 response could have potentially improved prevention measures and decreased mortality. Different knowledge systems widen the conceptual understanding and provide diverse context-specific actions to respond. All this points to the need to shift and recentre the biography and geography of knowledge based on the recognition that Africa is a “legitimate historical unit of analysis and epistemic site from which to interpret the world while at the same time globalizing knowledge from Africa. Such a move constitutes epistemic freedom as that essential prerequisite for political, cultural, economic, and other freedoms” [20] (p. 4). Put differently, there is the need to “shift the geo- and body-politics of knowledge from its foundation in Western imperial history of the past five centuries, to the geo- and body-politics of people, languages, religions, political and economic conceptions, subjectivities, etc., that have been racialized (that is, denied their plain humanity)” [22] (p. 13). This is why the focus of this paper is on the efficacy of IKS in an African and South African setting in responding to the COVID-19 pandemic. This intersects with the decolonial lens deployed in this paper, demonstrating that the power politics that elevate Euromodernity and its knowledge as the primary response to pandemics like COVID-19 are misplaced and need to be unsettled and troubled. 

## 4. Indigenous Knowledge Systems

Indigenous communities, present in approximately ninety countries around the globe, account for about five percent of the world’s population [23,24]. Over generations, these communities have leveraged IKS for health, wellbeing, and strengthening community resilience. Moreover, IKS encompass a broad spectrum of knowledge domains like agriculture, education, training, environmental conservation, and health [6], making it a versatile tool for indigenous communities in managing their socio-economic and ecological wellbeing. The characteristics of IKS include cost-effectiveness, cultural context, creative experiential knowledge, social inclusion, dynamism, support for local decision-making, and transmission by oral tradition from one generation to another [6]. To date, IKS have been framed as an “add-on” to Euro-western scientific knowledge, sidelined in mainstream healthcare governance, and neglected in policy and practice. The WHO has disregarded IKS, citing concerns over their validity [6]. Yet, there is complementarity between IKS and Euro-western scientific knowledge. IKS bring additional perspectives, foci, and aims concerning healthcare due to geography and socio-cultural context and is, therefore, indispensable for healthcare policies and programmes, given that we might experience more pandemics in the future and IKS have played a prominent role in facilitating responses to past epidemics and pandemics [6]. Nonetheless, the integration of IKS into health plans has been slow and inconsistent, with local communities only recently recognized as pivotal actors in health policy. However, there is a lack of sufficient knowledge regarding the deployment of IKS to respond to COVID-19, as well as answers to related questions such as what IKS is being deployed, where, why, for whom, and by whom, especially in the African context. Hence, this study explores these questions in the UKDM. Understanding the potential role of IKS in addressing the challenges posed by COVID-19 could offer innovative and contextually relevant strategies for response and recovery. 

## 5. Study Setting and Methodological Approach 

**Figure 1:**
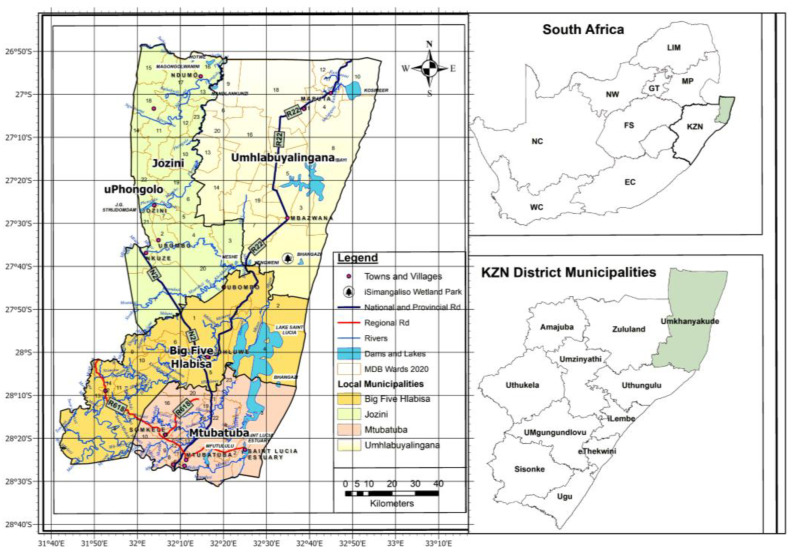
uMkhanyakude District Municipality within South Africa (Source: author, 2024).

This paper is based on research concentrating on the UKDM, identified as KwaZulu-Natal’s (KZN) second-largest district [25]. The UKDM is divided into four Local Municipalities (LMs): Mtubatuba, the Big Five Hlabisa, Jozini, and uMhlabuyalingana [26] (Figure 1). These LMs comprise 71 wards and 151,245 households with a population totalling 689,090 [26]. The demographic distribution within this population is characterized by a majority of females (53%) in contrast to males (47%). Furthermore, approximately 99.3% of the population are black Africans [26], and 98% of the population reside in rural areas [27]. Agriculture largely shapes the socio-economic and cultural context of the UKDM populace [25]. About 95% of the rural population within the district depend on small-scale agriculture, social security grants [28,29], and remittances from migrant workers to survive [29]. 

The UKDM is couched in a history of marginalization, which has significantly impacted its basic service delivery infrastructure. This inadequacy in services can be traced back to the apartheid era, during which the UKDM was categorized as part of the former homelands, leading to prolonged neglect by the apartheid regime [30]. Moreover, political struggles over power continue to impede progress in the provision of basic services [31]. Healthcare services within the district are delivered through clinics and regional hospitals. This is in addition to the traditional health system comprised of herbalists and traditional healers, who are generally highly regarded [32] and hold a significant cultural role and influence in provinces such as KwaZulu-Natal [33]. The UKDM was selected as a case study because it is a predominantly rural district adjudged amongst the poorest districts in KZN [34,35]. The district faces significant challenges due to inadequate health facilities and the high transport costs associated with accessing healthcare [32,36], rendering its communities particularly susceptible to COVID-19. Traditional practices and communal living arrangements amplify this vulnerability. Given the district’s extensive geographical spread, a multistage sampling technique was employed to adequately represent the diversity within the UKDM. This process involved stratifying the UKDM into its four constituent LMs. Subsequently, one village from each LM was purposively selected—Umhlabuyalingana LM (Nhlamvu), Jozini LM (Makhonyeni), Big Five Hlabisa LM (Nyathini), and Mtubatuba LM (Dukuduku). This ensured broad coverage across different agro-ecological zones and facilitated a more encompassing examination of the UKDM.

Qualitative methods (semi-structured face-to-face interviews and document analysis) were used to facilitate an in-depth inquiry into the coloniality of COVID-19 responses and the use of IKS to respond to the pandemic. Qualitative research refers to “an array of interpretive techniques which seek to describe, decode, translate and otherwise come to terms with the meaning, not the frequency, of certain more or less naturally occurring phenomena in the social world” [37] (p. 520). Through a qualitative approach, a researcher can obtain a detailed contextualized perspective based on respondents’ voices [38]. The researchers purposively sampled UKDM community members to capture different perspectives to inform an understanding of the research problem [39]. The inclusion criteria required respondents to be household heads aged 18 years or older, impacted by COVID-19, and knowledgeable about indigenous responses to COVID-19.

In qualitative research, there are no stringent rules for determining the appropriate sample size. The sample size was determined by the level of information saturation, resulting in a sample of 20 respondents. A small sample size is justified because qualitative research generates detailed and nuanced understanding and does not test hypotheses [40]. Before commencing data collection, permission to conduct research in the study sites was obtained from the Gatekeepers (traditional leaders). The researchers facilitated the interviews in isiZulu or English, depending on the respondents’ preferences. This linguistic flexibility ensured that respondents could express their views and experiences in the language in which they felt most comfortable, thereby enhancing the quality and authenticity of the data collected. The interviews were conducted between June and July 2023 at the respondents’ residence and lasted 25 to 45 min on average.

Through purposive sampling, various types of documents (including academic journal articles, public documents, and grey literature) relating to IKS, COVID-19, and knowledge decolonization were sourced and reviewed via desktop study (using electronic databases and Google) [41]. Similar to the interviews, the document analysis reflects people’s beliefs and perspectives [42]. However, unlike the interviews, the document analysis provides stable data that the researcher has not influenced and that would otherwise require considerable effort and time to collect [42]. These documents were accessed to contextualize the research findings and facilitate triangulation with the data acquired from the interviews. Reflexive thematic analysis was employed to analyse the qualitative data as it is one of the approaches based solely on qualitative methods and fully embraces qualitative research values [42,43]. This data analysis technique identifies, analyses, and reports themes within data in great detail [44]. It enables the researcher to examine multiple perspectives, note similarities and differences, and identify new insights [45]. In this approach, the researcher’s subjectivity is a valuable analytic resource as his or her knowledge, experiences, feelings, and social position (e.g., race, gender, class) influence the data interpretation process [43]. Moreover, this approach empowers the researcher to comprehensively address diverse subjects via interpretations rather than just synthesizing data [43]. 

## 6. COVID-19 Responses, Environmental Stress, and Community Resilience in the UKDM

As an entry point to the data analysis, there is a need to provide a contextual background that, in South Africa and in the UKDM, in particular, an authoritarian political culture was maintained, shaped by the historical colonial legacy that influenced the response to the pandemic without considering the diverse realities of communities. Like other governments, the South African government responded to the pandemic as a law-and-order problem and failed to address vulnerability based on context, overshadowing the nuanced realities of local communities. This misstep manifested in harsh enforcement tactics by law enforcement agencies, including soldiers and police, under the guise of implementing preventive and control measures such as lockdowns and social distancing protocols. Such measures, predicated on a lack of trust in individuals’ capacity for self-regulation, culminated in widespread brutality, epitomized by the tragic demise of Collins Khosa [46]. On 10 April 2020, members of the South African National Defence Force (SANDF) entered Khosa’s premises in Alexandra, Gauteng, accused him of violating COVID-19 lockdown regulations, and subsequently assaulted him. Three hours after their departure, Khosa succumbed to his injuries [47,48]. Moreover, between 26 March and 5 May 2020, there were 10 reported deaths and 280 assaults across South Africa related to COVID-19 operations conducted by the SANDF and the South African Police Service (SAPS) [49]. This suggests that a more effective mitigation strategy, avoiding loss of lives and violence, would have been the formulation of confinement regulations grounded in local autonomy and self-determination. The decoloniality perspective highlights the missed opportunity to leverage the geographical remoteness of study sites, which could have provided a buffer during the initial stages of the pandemic. Such a buffer period could have been strategically utilized to develop and implement preparedness measures that are both contextually relevant and sensitive to the local socio-political and environmental landscape [50]. 

The UKDM respondents shared that the COVID-19 pandemic coincided with a drought period. This further challenged the UKDM communities that were already dealing with draconian prevention and control measures. These measures heightened vulnerability, particularly for those reliant on natural resources and agriculture for sustenance, which is a significant proportion of the UKDM population. The economic marginalization of the UKDM community members underscores their dependence on agricultural practices. One respondent recounted their experience as part of the *Masihambisane* agricultural co-operative during the pandemic, revealing how the inability to convene impacted their farming negatively. This underscores the communal nature of African societies and how their way of life underwent a profound transformation due to the stringent measures adopted by the government. Additionally, misinformation regarding the transmission of COVID-19—such as the unfounded belief that the virus could be contracted simply by being outside through the air—led to significant behavioural changes, including the decision by some respondents to halt agricultural activities. However, COVID-19 primarily spreads from person to person through respiratory droplets that are expelled when an infected person talks, sneezes, coughs, laughs, sings, eats, or breathes. This underscores the critical role of misinformation in shaping community responses to the pandemic and suggests that integrating traditional leaders and healers into the decision-making process could have promoted more accurate and culturally resonant health communications. The fields, once a source of safety and livelihood, paradoxically transformed into hazardous spaces where contracting COVID-19 and encountering law enforcement brutality became concerns. This situation reflects the intricate ways in which pandemic response measures, environmental challenges, and socio-political structures converge to reshape community landscapes and livelihoods.

### 6.1. Vaccine Scepticism and the Resurgence of Indigenous Knowledge in COVID-19 Response 

Upon the initiation of the vaccination programme by the South African government, the response among the study respondents was mixed. Some respondents embraced vaccination, but there was resistance from others who cited concerns about being used as test subjects. This resistance was fuelled by comments from French doctors suggesting that the vaccines would be tested on Africans, which in turn cast doubt on the motives behind vaccine donations from Western countries [8]. Such scepticism was not confined to donated vaccines alone but extended to those procured for distribution, with many individuals displaying hesitancy towards inoculation. This scepticism seemed validated when subsequent research linked the AstraZeneca vaccine, one of the administered vaccines, with a risk of blood clots [8].

Amidst the backdrop of conspiracy theories and growing mistrust towards vaccination, the respondents of this study increasingly turned to IKS to counter the COVID-19 pandemic. The recourse to IKS encompassed the utilization of traditional practices such as steam inhalation and the preparation of herbal concoctions, leveraging the trust, widespread acceptance, and accessibility of these methods within their communities. Notably, ingredients such as lemon bush and eucalyptus leaves were commonly used for steam inhalation. Others employed incense bush and African wormwood (*umhlonyane*) for the same purpose. Concoctions were made from a variety of substances, including lemon bush, eucalyptus, garlic, ginger, and honey. Additionally, some practices involved the use of lemon bush for colon cleansing and the induction of vomiting using rough salt. The reliance on such practices, despite the absence of scientific validation in accordance with WHO standards, reflects a deep-seated confidence in their efficacy, deriving from their long-standing usage and perceived effectiveness in preventing or treating COVID-19, thereby constituting a form of “experimental knowledge”.

The use of *umhlonyane* and similar concoctions within the UKDM finds support from other scholars e.g., [4], who propose that these remedies might not necessarily cure COVID-19 but could serve as preventive measures or offer support for mild cases. These practices, underpinned by IKS, highlight an adaptive response to the pandemic, where community members, including one who emphasized the benefits of exercise and dietary adjustments for enhancing immunity, actively engaged in health-promoting behaviours. The widespread acceptance and application of such herbal remedies, as evidenced in South Africa and countries like Zimbabwe, reflect their significance within a broader African context. Similarly, in Brazil, Mondardo [51] reported the use of indigenous herbal preparations to alleviate COVID-19 symptoms. Haokip [52] notes that hill communities in Northeast India employed centuries-old epidemic control measures, such as lockdowns, quarantine, isolation, and disinfection, often more stringently than government mandates. For instance, indigenous groups in the highlands used their traditional knowledge to impose self-lockdowns, block main roads, and establish temporary footpaths to connect villages, allowing movement only after the priest declared the lockdown over. In Sohtyllang, Southwest Khasi hills, 770 bamboo quarantine centres were set up for returning young migrants, while the state ran 17 centres. When domestic flights resumed on 25 May 2020, the state mandated 14 days of institutional quarantine, but community organizations in Churachandpur and Kangpokpi districts extended this period by an additional 14 days based on their experience of past epidemics. The reliance on indigenous remedies and other epidemic control measures, fostered by generations of empirical use, challenges the predominant global health narratives that prioritize pharmaceutical interventions, such as vaccines, over IKS and practices. However, those in power advocated for vaccines. This raises pertinent questions about the universal applicability of WHO efficacy standards and the marginalization of indigenous health practices in the global health discourse. The insistence on the WHO standards for validating the efficacy of local remedies not only overlooks the contextual relevance and accessibility of these practices but also perpetuates a form of epistemic injustice. This exclusion from the global discourse on health interventions suggests a need for a more inclusive approach that acknowledges the value of diverse IKS in addressing health crises. Such an approach would not only enrich global health strategies with various perspectives but also empower communities by recognizing and integrating their indigenous practices into broader health responses.

### 6.2. Mechanics Required for Indigenous Knowledge Systems to Be Promoted in Policy and Programmes 

The reliance on external knowledge and solutions is costly for resource-constrained Africa, hence the need to mobilize indigenous epistemologies [6]. One of the respondents noted a concerning trend where the custodians of IKS, predominantly elders, exhibit a diminishing proclivity towards disseminating IKS. This reluctance is compounded by a disinterest among the youth, a phenomenon attributed to the residual impacts of colonialism, which valorizes Western epistemologies over indigenous ones. The declining transmission of IKS, was poignantly highlighted by a respondent’s observation “*abasekho omkhulu nogogo abantu abanolwazi*”, which translates to “elders are no longer alive.” This underscores a critical need for strategies to facilitate the preservation and transfer of knowledge across generations to safeguard the future of IKS. This situation is dire, with the threat to the continuity of IKS intensified by the passing away of its custodians, a situation further worsened by the impacts of the COVID-19 pandemic. Such developments underscore the vulnerability of IKS, emphasizing the vital role of fostering multigenerational housing as a means to preserve and transmit this invaluable knowledge.

The scepticism of the youth towards IKS, influenced by their education in Western paradigms, further challenges the appreciation and preservation of IK, relegating it to a secondary status behind Western scientific knowledge. To counteract this trend, it is imperative to dismantle the remnants of colonial thought patterns and affirm the value of IKS, tailored to address Africa’s distinct challenges and realities [6]. A key insight from one of the respondents is that Western knowledge, although comprehensible, is challenging to access due to the need for resources like books or Internet data, which are not readily available to everyone. In contrast, the accessibility of IKS thrives on oral transmission and practical application within daily life. This intrinsic mode of knowledge transmission aligns with African traditions, emphasizing the role of IKS not merely as a knowledge system but as a lived epistemological practice.

In the context of the COVID-19 pandemic, the importance of a coordinated regional approach that leverages trusted traditional leaders and healers for information dissemination became evident. This approach not only facilitates effective communication but also underscores the need for integrating IKS into formal education systems, including university health curricula. Such integration, coupled with the promotion of multilingualism, would enhance the comprehension, preservation and translation of IKS, thereby broadening its accessibility and application. By embedding IKS within educational curricula and fostering their development through communal practices, a dynamic and communal epistemology can be nurtured, ensuring that IK remains a vital component of Africa’s socio-cultural and epistemological fabric. 

## 7. Conclusions

In this paper, we sought to demonstrate that the response to COVID-19 in an African and South African context was driven by coloniality in that the IKS of local communities within the UKDM were marginalized and trivialized. This is consistent with Euromodernity, which valorizes Western canon as knowledge and everything else as myth or lies. This is where the problem lies, because knowledge can only be universal if it is pluriversal [53]. This means that Western science is not universal, and the fact that it has been allowed to occupy this position must be questioned. The COVID-19 pandemic response missed the opportunity to recognize and incorporate the valuable contributions of IKS to health and wellbeing. This oversight was largely due to the prevailing colonial assumptions that prioritize Western knowledge over indigenous practices. Our analysis reveals that, despite the pervasive coloniality characterizing the pandemic’s response in the UKDM, local communities were able to leverage their IK. Differently stated, people were able to use approaches such as steaming and traditional herbs and medicine to respond to COVID-19, and this yielded positive impacts, as it was shown that many people survived the pandemic through such medication. To this degree, IKS are efficacious in tackling pandemics like COVID-19. For this cause, “what Africans must be vigilant against is the trap of ending up normalising and universalising coloniality as a natural state of the world. It must be unmasked, resisted, and destroyed because it produced a world order that can only be sustained through a combination of violence, deceit, hypocrisy, and lies” [15] (p. 11). This paper, therefore, not only highlights the resilience and resourcefulness of the UKDM communities in navigating the pandemic through indigenous practices but also calls for a critical examination of the structures that continue to marginalize and invalidate non-Western forms of knowledge. In doing so, it advocates for a more inclusive, pluriversal approach to knowledge that values and integrates the diverse epistemologies inherent within global communities, particularly in the face of shared challenges like pandemics.

## Data Availability

The datasets generated and/or analyzed during the study are not publicly available due to privacy and confidentiality concerns related to participant information, but they are available from the corresponding author upon reasonable request.
